# Associations between pre-pandemic housing insecurity and reports of anxiety and depression in the early months of the COVID-19 pandemic: an analysis of the *All of Us* Research Program cohort

**DOI:** 10.1186/s12889-025-24535-w

**Published:** 2025-10-01

**Authors:** Giselle Routhier, Andrea R. Titus, Yao Xin, Binyu Cui, Stephanie H. Cook

**Affiliations:** 1 Department of Population Health, NYU Grossman School of Medicine, New York, NY USA; 2 Department of Biostatistics, NYU School of Global Public Health, New York, NY USA

**Keywords:** Housing insecurity, COVID-19, Anxiety, Depression, Mental health

## Abstract

**Background:**

Housing insecurity is an important social determinant of health that has been linked to poor mental health. The beginning of the COVID-19 pandemic marked a time of major upheaval and has been linked to worsening experiences with anxiety and depression. It is not well understood how pre-pandemic housing insecurity may be associated with trajectories of anxiety and depression in the early months of the COVID-19 pandemic.

**Methods:**

Using data from the NIH *All of Us* Research Program, we estimated the correlation between pre-pandemic housing insecurity and repeated measures of anxiety and depression symptoms between May and July 2020. We combined data from baseline surveys and the COVID-19 Participant Experience Survey (COPE) and used generalized linear models with a logit link to estimate results.

**Results:**

Our sample included 37,535 participants. Those who reported housing insecurity prior to the start of the pandemic were significantly more likely to report moderate-to-severe symptoms of anxiety (AOR 1.630, *p* < .001) and depression (AOR 1.877, *p* < .001) across all months. Trajectories of anxiety and depression symptoms did not differ between May and July 2020 for those who reported housing insecurity versus those who did not.

**Conclusions:**

This study examined how experiences and trajectories of anxiety and depression symptoms differed by housing insecurity status among a novel large national sample. Experiencing housing insecurity prior to the start of the COVID-19 pandemic was associated with a greater likelihood of moderate-to-severe anxiety and depression from May to July 2020, and this difference was consistent over time.

**Supplementary Information:**

The online version contains supplementary material available at 10.1186/s12889-025-24535-w.

## Background

Housing insecurity is a large and growing problem in the United States [1], which can manifest in multiple ways, including unaffordability, poor housing conditions, crowding, evictions, or homelessness [2]. Prior to the start of the COVID-19 pandemic, nearly 23 million renters experienced some form of housing insecurity [3] and 580,000 people experienced homelessness on any given night [4]. Housing insecurity is a well-known social determinant of health [5], which has been linked to poor mental health outcomes, including depression and anxiety [6–10]. Because housing insecurity also disproportionately impacts Black, Hispanic, and indigenous persons [3], it is also relevant to understanding and addressing health equity.

Research on the impact of the COVID-19 pandemic on mental health has shown an association between the onset of the pandemic and worsening mental health – including high rates of symptoms of anxiety, depression, post-traumatic stress disorder, and psychological distress, among other outcomes – compared to pre-pandemic levels [11–14]. While the pandemic had wide-ranging effects on the general population, it may have exacerbated the impact of preexisting health-related social needs, such as housing insecurity, on mental health outcomes. For example, it is possible that individuals facing housing insecurity may have experienced more severe economic impacts and/or greater risk for infection (e.g., through exposure in congregate shelter settings or crowded housing) leading to greater struggles with mental health [15–18]. In addition, prior research suggests that these dynamics may have been particularly pronounced in the early months of the pandemic, when economic stimulus payments and major housing initiatives (such as rental assistance and eviction moratoriums) were either only just beginning or had not yet been implemented [14, 19]. For example, a cross-sectional study of 3,200 adult women in the United States found that women with pre-pandemic housing insecurity had higher odds of experiencing depression, anxiety, and posttraumatic stress in the early phase of the pandemic [14]. Understanding the impacts of major public health or other crises on mental health outcomes among individuals experiencing housing insecurity or other forms of economic instability is critical to developing policies that mitigate the potential negative impacts of these events on health and health equity.

To our knowledge, no studies have explored differences in the trajectories of depression and anxiety in the early months of the COVID-19 pandemic for those with secure and insecure housing prior to the start of the pandemic. This study seeks to understand how housing insecurity is associated with symptoms of anxiety and depression in the early months of the COVID-19 pandemic within a large national sample and whether trajectories of reported symptoms differed between those with secure and insecure housing between May and July 2020. Understanding how pre-pandemic health-related social needs, such as housing insecurity, are associated with mental health outcomes after the pandemic began is important for understanding how crisis response policies should be targeted, as well as for informing broader policies to address social determinants of health, such as housing.

## Methods

### Aims

The aim of this study was to measure associations between pre-pandemic housing insecurity and reports of anxiety and depression symptoms in the early months of the pandemic and to measure whether changes in reports of anxiety and depression differed between May and July 2020 for those experiencing housing insecurity versus those who did not.

### Data and sample

We conducted secondary data analyses using data from the National Institutes of Health (NIH) *All of Us* Research Program (All of Us), a national longitudinal cohort study that combines data from surveys, electronic health records, bio samples, and other sources [20]. The *All of Us* Research Program is a large-scale effort with a particular emphasis on recruiting populations that have been historically underrepresented in biomedical research, including racially minoritized populations. All authors are registered users with the All of Us researcher workbench.

We combined data from the baseline surveys (“The Basics” survey) from participants enrolled through May 2020 and the first three waves of the COVID-19 Participant Experience Survey (COPE), which were administered in May, June, and July 2020. The Basics survey is administered to all participants at baseline and includes a range of demographic information, including information on housing status. The COPE survey was administered as an optional survey at multiple time points during the COVID-19 pandemic and includes information about participants’ mental and physical health, including the Generalized Anxiety Disorder Questionnaire (GAD-7) and the Patient Health Questionnaire (PHQ-9), our outcome variables of interest.

We included all participants who had baseline survey data prior to the first COPE survey wave. The majority (96%) of participants in our sample had baseline survey data from 2018 onward.

### Outcomes and exposure variables

The GAD-7 is a validated self-report screening tool that measures symptoms of anxiety, with scores ranging from 0 to 21 [21]. The PHQ-9 is a similarly self-reported, validated screening tool for depression, with scores ranging from 0 to 27 [22]. Both the GAD-7 and PHQ-9 are included in the first three waves of the COPE survey. We constructed dichotomous variables from the reported GAD-7 and PHQ-9 scores to categorize participants who reported moderate or severe symptoms of anxiety or depression, versus those who did not. A score of 10 or higher on the GAD-7 indicates moderate or severe symptoms of generalized anxiety disorder [23] and a score of 15 or higher on the PHQ-9 indicates moderately severe or severe symptoms of depression [24].

Housing insecurity, our exposure variable of interest, was self-reported by participants in the Basics survey. Upon enrolling in the study, participants were asked: “In the past 6 months, have you been worried or concerned about NOT having a place to live?” Participants who answered “yes” were considered to have experienced housing insecurity.

### Covariates

We included multiple potentially confounding variables in our models from the baseline interview, including age (continuous), race/ethnicity (White, Black, Asian, Hispanic, Other), gender identity (man, woman, other), sexual orientation (straight, gay, lesbian, bisexual, other), education (advanced degree, college graduate, some college, high school or GED, less than high school), yearly income (75k or more, 50-75k, 25-50k, less than 25k, unknown), employment (employed, self-employed, homemaker, student, out of work less than one year, out of work one or more years, retired, unable to work, other), marital status (married, never married, divorced/separated, living with partner, widowed), years at current address (less than 1 year, 1–2 years, 3–5 years, 6–10 years, 11–20 years, more than 20 years), housing tenure (owner, renter, other), general physical health (poor, fair, good, very good, excellent), general mental health (poor, fair, good, very good, excellent), and insurance status (insured, insured with Medicaid, not insured). All confounding variables are listed in Table 1.

### Analytic methods

To estimate the correlation between repeated measures of anxiety disorder or depression and baseline housing security, we fit generalized linear models with a logit link, using a generalized estimating equations (GEE). Those who had a baseline value for housing insecurity, as well as the GAD-7 and PHQ-9 scores in the first wave of the COPE survey, were used as the estimation sample. This included 37,535 participants, among which 17,919 and 15,572 individuals completed the second and the third wave, respectively. In the sample, 12,151 participants completed two waves and 10,670 completed all three waves. GEE statistical models can handle large amounts of missing values and offer parameter estimates for the population average of the repeatedly measured outcomes.

Our model included the exposure variable of interest, housing insecurity, and adjusted for all confounding variables, as well as survey wave. We also included an interaction between housing insecurity and wave to assess whether trajectories of GAD-7 and PHQ-9 scores differed for those who reported housing insecurity versus those who did not. Additionally, we conducted a sensitivity analysis which restricted the sample to participants who were enrolled in All of Us and completed the baseline survey between January and May 2020 (*n* = 6,325), to assess whether results changed when we limited the sample to individuals with more recent measures of housing insecurity, proximal to the onset of the pandemic. Statistical analyses were conducted with R 4.2.2 embedded in the All of Us data workbench. The *All of Us* Research Program uses a data passport model for data access. IRB approval was not needed.

## Results

Our sample included 37,535 participants, representing 12% of the full All of Us sample at the time of analysis. Baseline demographic characteristics and confounders are included in Table 1. Overall, sample participants reported high incomes and high rates of education and employment. The median age at enrollment was 60 (IQR 45–69). Most of the participants identified as women (65%), White (84%), were married (60%), and straight (91%). About 20% of the sample were renters. Most (84%) participants reported at least “good” physical and mental health and the vast majority (98%) had health insurance. The percentage of participants reporting pre-pandemic housing insecurity was 6.7%. In the first wave, 14% of participants reported symptoms of moderate-to-severe anxiety and 6.4% reported symptoms of moderate-to-severe depression. In the third wave, 12% reported symptoms of moderate-to-severe anxiety and 5.7% reported symptoms of moderate-to-severe depression. Among sample participants who reported housing insecurity, 36% reported symptoms of moderate-to-severe anxiety in the first wave and 34% in the third wave. 25% reported moderate-to-severe symptoms of depression in the first wave and 23% in the third wave. Unadjusted GAD-7 and PHQ-9 scores by wave and housing insecurity status are presented in Table 2.

**Table 1:** Demographic characteristics at baseline in the Estimation sample (*n* = 37,535)

Variable	*n* (%)
Housing Insecurity	
No	35,015 (93)
Yes	2,520 (6.7)
Age (at consent)	
Median (IQR)	60 (45, 69)
Range	19, 115
Gender	
Woman	24,479 (65)
Man	12,871 (34)
Other	185 (0.5)
Sexual Orientation	
Straight	34,050 (91)
Bisexual	1,299 (3.5)
Gay	1,101 (2.9)
Lesbian	579 (1.5)
Other	506 (1.3)
Race/Ethnicity	
White	31,491 (84)
Black	1,748 (4.7)
Hispanic	1,029 (2.7)
Asian	2,217 (5.9)
Other	1,050 (2.8)
Education	
Advanced Degree	15,010 (40)
College Graduate	12,106 (32)
College One to Three	7,931 (21)
Twelve or GED	2,259 (6.0)
Less than high school	229 (0.6)
Annual Household Income	
75k or greater	20,190 (54)
50k – 75k	5,803 (15)
25k – 50k	5,486 (15)
Less than 25k	3,465 (9.2)
Unknown	2,591 (6.9)
Employment Status	
Employed	16,503 (44)
Self-Employed	1,961 (5.2)
Homemaker	888 (2.4)
Student	593 (1.6)
Out of Work Less Than One Year	501 (1.3)
Out of Work One or More Years	512 (1.4)
Retired	11,402 (30)
Unable to Work	1,550 (4.1)
Other	3,625 (9.7)
Marital Status	
Married	22,346 (60)
Never Married	6,261 (17)
Divorced or separated	5,015 (13)
Living with Partner	2,092 (5.6)
Widowed	1,821 (4.9)
Years Lived in Current Address	
Less than 1	4,200 (11)
1 to 2	4,793 (13)
3 to 5	6,089 (16)
6 to 10	4,941 (13)
11 to 20	7,399 (20)
More than 20	10,113 (27)
Own or Rent Current Home	
Own	26,911 (72)
Rent	8,694 (23)
Other	1,930 (5.1)
General Physical Health	
Poor	946 (2.5)
Fair	4,895 (13)
Good	11,891 (32)
Very Good	14,795 (39)
Excellent	5,008 (13)
General Mental Health	
Poor	563 (1.5)
Fair	3,193 (8.5)
Good	8,687 (23)
Very Good	14,834 (40)
Excellent	10,258 (27)
Insurance	
No	701 (1.9)
Yes, not Medicaid	36,139 (96)
Yes, Medicaid	695 (1.9)

1 Median (IQR) or Frequency (%), unless otherwise specified

**Table 2:** Symptoms of moderate-to-severe anxiety and depression by wave and baseline housing insecurity status

Variable	Wave 1, *N* (%)	Wave 2, *N* (%)	Wave 3, *N* (%)
Full sample			
General Anxiety Disorder-7			
No	32,200 (86%)	15,707 (88%)	13,757 (88%)
Yes	5,335 (14%)	2,212 (12%)	1,815 (12%)
Patient Health Questionaire-9			
No	35,130 (94%)	16,897 (94%)	14,689 (94%)
Yes	2,405 (6.4%)	1,022 (5.7%)	883 (5.7%)
No housing insecurity			
General Anxiety Disorder-7			
No	30,595 (87%)	15,003 (89%)	13,152 (90%)
Yes	4,420 (13%)	1,827 (11%)	1,509 (10%)
Patient Health Questionaire-9			
No	33,237 (95%)	16,064 (95%)	13,987 (95%)
Yes	1,778 (5%)	766 (5%)	674 (5%)
Housing insecurity			
General Anxiety Disorder-7			
No	1,605 (64%)	704 (65%)	605 (66%)
Yes	915 (36%)	385 (35%)	306 (34%)
Patient Health Questionaire-9			
No	1,893 (75%)	833 (76%)	702 (77%)
Yes	627 (25%)	256 (24%)	209 (23%)

Fully-adjusted GEE model results are presented in Table 3, displayed as adjusted odds ratios. After adjusting for all potential confounders and waves, participants who reported housing insecurity prior to the start of the pandemic were significantly more likely to report moderate-to-severe symptoms of anxiety (AOR 1.630, *p* <.001) and depression (AOR 1.877, *p* <.001) across all survey waves. We found that anxiety scores decreased significantly between wave 1 and wave 3 on average for all participants, while depression scores did not change between waves. Examining the interaction between housing insecurity and wave, we found that trajectories of anxiety and depression did not differ for those who reported housing insecurity versus those who did not. Results were consistent for the sensitivity analysis subsample of respondents who reported housing insecurity in 2020. Predicted probabilities of reporting moderate to severe symptoms of anxiety or depression in the fully adjusted models were similar to the unadjusted values (Appendix 1).

**Table 3:** Measures of anxiety and depression for fully adjusted analytic models, odds ratios and confidence intervals

	GAD-7	PHQ-9
Housing Insecurity = yes	1.630 (1.462–1.814)***	1.877 (1.650–2.137)***
Wave	0.926 (0.903–0.950)***	0.973 (0.937–1.009)
Housing Insecurity*Wave	1.033 (0.959–1.113)	0.969 (0.886–1.061)

***p <.001

## Discussion

Overall, our analysis showed that people experiencing housing insecurity before the pandemic began were more likely to experience moderate-to-severe symptoms of anxiety and depression during the early months of the COVID-19 pandemic than those with secure housing. The trajectories of reported anxiety and depression between May and July 2020 were similar for those with housing insecurity versus those with stable housing, with consistently higher reported anxiety and depression scores at each wave for people with pre-pandemic housing insecurity.

Our study specifically examined how experiences and trajectories of anxiety and depression symptoms differed by housing insecurity status among a novel large national sample, and thus expands on existing literature. Expectedly, people who reported housing insecurity were more likely to experience moderate-to-severe anxiety and depression in the early months of the COVID-19 pandemic, and this trend was stable over time. This finding aligns with prior studies, which have documented high rates of anxiety and depression symptoms among individuals experiencing housing insecurity during the pandemic [15, 16, 19]. Differences in mental health outcomes may reflect existing disparities in place prior to the start of the pandemic, rather than being a consequence of the pandemic itself. Since we did not have the same measures of anxiety and depression in the baseline survey, we were not able to compare pre-and post-pandemic measures. However, we adjust for baseline measures of general mental health, and we did have multiple waves of depression and anxiety measures between May and July 2020, which allowed us to examine trajectories of mental health outcomes during a period of particularly rapid social and economic change. Of note, individuals experiencing housing insecurity did not experience differential trajectories in reports of anxiety and depression symptoms compared with those who did not experience housing insecurity, but rather experienced sustained higher rates of anxiety and depression from May to July 2020.

These findings contribute to the broader understanding of housing insecurity, mental health, and the COVID-19 pandemic, with valuable implications for policy. Future response policies should consider how existing social determinants of health might be exacerbated, or remain elevated, in the immediate crisis-response stage and explore how to best meet the needs of vulnerable populations. Notably, the three survey waves in our study collected data after the federal government issued the first stimulus checks, but before they fully implemented large-scale efforts to prevent evictions, homelessness, and worsening housing insecurity, such as the CDC’s nationwide eviction moratorium and emergency rental assistance programs [25]. Future research should aim to examine the effects of targeted policies on mental health outcomes for people experiencing housing insecurity and whether such policies lessened the gap between experiences of anxiety and depression among people with and without housing insecurity.

Our findings also broadly highlight the need for additional research on the impact of housing insecurity on mental health, including examining differences by race, ethnicity, gender, and other sociodemographic characteristics which are known to be associated with housing insecurity, as well as examining differences over time. Housing insecurity is not a static experience and research designs should consider adopting longitudinal methods to assess housing insecurity, particularly to better capture changes in the relationship between housing status and health as well as to better estimate the bidirectional nature or causal pathways between housing insecurity and health outcomes.

### Limitations

Our study has several limitations. First, although we included pre-pandemic reports of general mental health as a confounding variable, we were unable to measure pre-pandemic reports of anxiety and depression with the GAD-7 and PHQ-9 scales, so are therefore unable to assess possible changes on participants’ scores. We are, therefore, unable to disentangle the extent to which differences in levels of depression and anxiety symptoms represented pre-existing differences between housing secure and insecure populations. Secondly, the three COPE waves cover a relatively short time period of three months. This may not have been enough time to measure meaningful changes in reports of anxiety and depression, although the early months of the pandemic represented a time of major upheaval and, therefore, were relevant to our study. Third, the majority of the participants were older, white, and reported high incomes. This is a reflection of the respondents that answered the COPE survey, which represented only a small portion of the full All of Us Research Program sample. As a result, our findings are not representative of nor generalizable to the United States population. Future studies should pursue related questions with a more diverse and representative sample. Additionally, future research should explore racial, ethnic, sexual, and gender differences in mental health trajectories and housing status, given known disparities in housing insecurity among these populations. Lastly, there may be unmeasured confounders that are not captured in All of Us - including other aspects of socioeconomic status or other aspects of physical health and wellbeing – which might impact the observed relationship between housing insecurity and symptoms of anxiety and depression.

## Conclusion

This study uses the *All of Us* Research program to examine the interaction between housing insecurity and experiences of anxiety and depression in the early months of the COVID-19 pandemic. All of Us presents a unique opportunity to examine national trends in health outcomes and social determinants of health. Findings are relevant for considering future pandemic response policies as well as ongoing efforts to collect data on housing insecurity and mental health outcomes.

## Supplementary Information

Supplementary material 1

## Data Availability

The dataset analyzed in this study is part of *the All of Us Research Program*. The *All of Us Research Program* employs a data passport model by which all researchers interested in using the data must be registered users and have an institutional data use and registration agreement in place. Registered users are able to access the data outlined in this study.

## Abbreviations

All of Us: All of Us research program
COPE: COVID-19 participant experience survey
GAD-7: Generalized anxiety disorder questionnaire
GEE: Generalized estimating equations
NIH: National institutes of health
PHQ-9: Patient health questionnaire
