# Supplementary material for: Associations between pre-pandemic housing insecurity and reports of anxiety and depression in the early months of the COVID-19 pandemic: an analysis of the All of Us Research Program cohort
Source: BMC Public Health. 2025 Oct 1;25:3266. doi: 10.1186/s12889-025-24535-w (PMC12487285; doi:10.1186/s12889-025-24535-w)

Appendix 1. Post-estimation predicted probabilities of GAD-7>10 (a) and PHQ-9>15 (b) at each wave, by housing insecurity status. Generated from adjusted GEE models.

a)


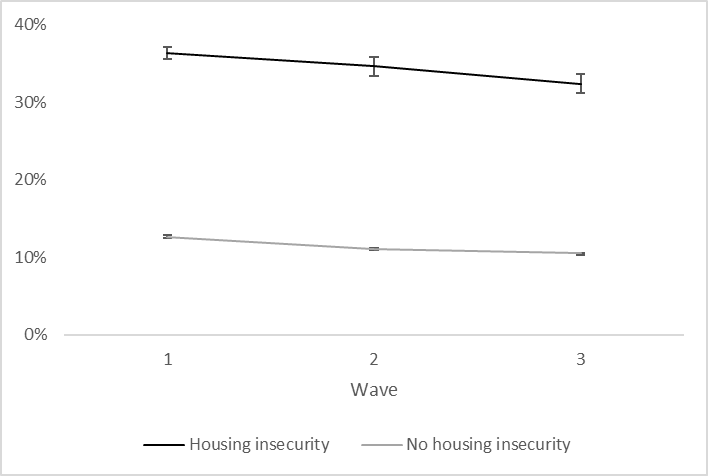


b)


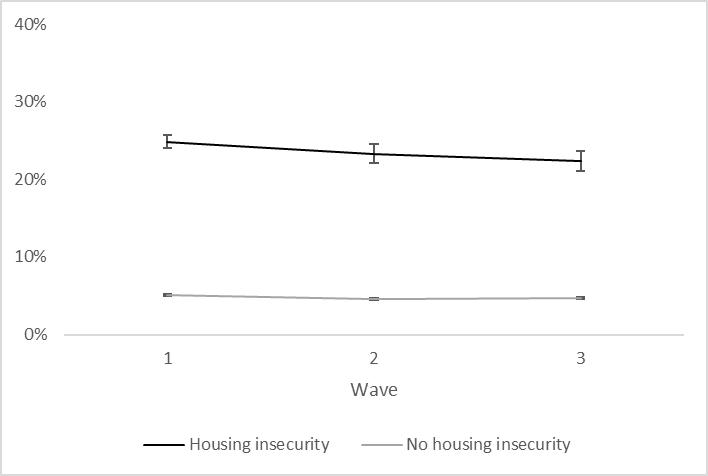

Supplement: Supplementary file 1 — Supplementary material 1 [file 12889_2025_24535_MOESM1_ESM.docx]
